# Main effect and epistatic QTL affecting spike shattering and association with plant height revealed in two spring wheat (*Triticum aestivum* L.) populations

**DOI:** 10.1007/s00122-021-03980-2

**Published:** 2022-03-20

**Authors:** Firdissa E. Bokore, Richard D. Cuthbert, Ron E. Knox, Heather L. Campbell, Brad Meyer, Amidou N’Diaye, Curtis J. Pozniak, Ron DePauw

**Affiliations:** 1 Swift Current Research and Development Centre, Agriculture and Agri-Food Canada, 1 Airport Road, P.O. Box 1030, Swift Current, SK S9H 3X2 Canada; 2 Department of Plant Sciences and Crop Development Centre, University of Saskatchewan, 51 Campus Drive, Saskatoon, SK S7N 5A8 Canada; 3 Advancing Wheat Technologies, 118 Strathcona Rd SW, Calgary, AB T3H 1P3 Canada

## Abstract

**Key message:**

A major QTL on chromosome arm 4BS was associated with reduced spike shattering and reduced plant height in coupling phase, and a second major QTL associated with reduced spike shattering was detected on chromosome arm 5AL in the same wheat variety Carberry.

**Abstract:**

Spike shattering can cause severe grain yield loss in wheat. Development of cultivars with reduced shattering but having easy mechanical threshability is the target of wheat breeding programs. This study was conducted to determine quantitative trait loci (QTL) associated with shattering resistance, and epistasis among QTL in the populations Carberry/AC Cadillac and Carberry/Thatcher. Response of the populations to spike shattering was evaluated near Swift Current, SK, in four to five environments. Plant height data recorded in different locations and years were used to determine the relationship of the trait with spike shattering. Each population was genotyped and mapped with the wheat 90 K Illumina iSelect SNP array. Main effect QTL were analyzed by MapQTL 6, and epistatic interactions between main effect QTL were determined by QTLNetwork 2.0. Correlations between height and shattering ranged from 0.15 to 0.49. Carberry contributed two major QTL associated with spike shattering on chromosome arms 4BS and 5AL, detected in both populations. Carberry also contributed two minor QTL on 7AS and 7AL. AC Cadillac contributed five minor QTL on 1AL, 2DL, 3AL, 3DL and 7DS. Nine epistatic QTL interactions were identified, out of which the most consistent and synergistic interaction, that reduced the expression of shattering, occurred between 4BS and 5AL QTL. The 4BS QTL was consistently associated with reduced shattering and reduced plant height in the coupling phase. The present findings shed light on the inheritance of shattering resistance and provide genetic markers for manipulating the trait to develop wheat cultivars.

**Supplementary Information:**

The online version contains supplementary material available at 10.1007/s00122-021-03980-2.

## Introduction

Seed shattering of wheat refers to loss of grain from the spike and to the loss of entire spikes (brittle rachis) from wheat standing in the field or prior to harvesting operations (Chang 1943; Porter 1959). We focus on the seed shattering trait as distinguished from the brittle rachis trait. Seed shattering results from disarticulation above the glume and brittle rachis results from disarticulation below the glume. Cultivated wheats are morphologically very different from ancestral forms. Domestication of wheat was based on mutations that resulted in a non-brittle rachis and kernel hullessness. Wheat genotypes that have a non-brittle rachis retain spikelets attached to the rachis, which means the spikelets do not disarticulate below the glume at maturity. The genes, *Non-brittle rachis 1 (btr1)* and *Nonbrittle rachis 2 (btr2),* are linked and located on chromosome 3AS (Pourkheirandish et al 2018). All cultivated wheat has a non-brittle spike that remains intact after maturity. Domestication of wheat resulted from other mutations that gave rise to hullessness and a non-brittle rachis. The free-threshing trait was attributed to the *Q* gene located on chromosome 5AL (Sears 1954). Simons et al (2006) used ectopic expression analysis of transgenic plants to demonstrate pleiotropic effects of the *Q* gene on traits such as glume shape and tenacity, rachis fragility, spike length, plant height, and spike emergence time which agreed with previously published cytogenetic analysis.

Shattering has long been known to cause yield losses in cereal crops such as oats, barley and wheat (Clarke 1981). Grain losses due to shattering are of economic importance and reported in different studies (Clarke 1981; Clarke and DePauw 1983; Porter 1959). Pincus (1931) conducted experiments in the western part of Serbia on the degree of shelling or shattering in newly developed Russian wheats and found some cultivars that shattered as much as 19.6% compared to 2.0 to 3.0% for other cultivars. Clarke and DePauw (1983) investigated the dynamics of shattering in maturing wheat and reported a shattering loss, expressed as a percentage of yield, that ranged from 3.2 to 17.3% over a three-week period beyond harvest ripeness (14.5% moisture wet weight basis). The cultivar Stoa expressed 2% shattering while Sumai3 exhibited 68% averaged over five environments in North Dakota (Zhang and Mergoum 2007).

Present day wheat cultivars are resistant to shattering compared with their ancestors, but some shattering still occurs due to varietal differences or weather conditions. Varietal morphological attributes including glume tenacity, kernel size, number of kernels per spikelet, awns, spike compactness, and environmental factors such as wind and humidity influence the ability of a cultivar to hold its grain in a recoverable position for a period of several weeks after maturity (Chang 1943; Clarke and DePauw 1983; Harrington and Waywell 1950). Shattering resistance is clearly of benefit during the post-maturity and pre-combine period and would be particularly desirable where direct combining is practiced (Clarke 1981). Mechanical threshability is a desired trait. There is a fine balance between a genotype holding the seeds firmly in the spike prior to harvest while during mechanical harvest the seed separates from the lemma and palea of the spikelet and glumes from the rachis without requiring expressive energy. Failure to separate, results in the retention of spike tissue, which may or may not be enclosing a kernel, that must be removed prior to milling. This spike tissue in a grain sample is classified as dockage by most international grain grading systems and reduces the value of the harvested grain.

Using inbred wheat lines derived from the population Ning7840/Clark and QTL analysis, Marza et al (2006) identified six loci associated with shattering resistance across chromosomes 4B, 5A, 6A, 6B and 7D. Using the same Ning7840/Clark population, Li et al (2016) identified an additional QTL on chromosome arm 2DS positioned in marker interval *Xwmc25.1 - Xgwm296.2*. Zhang and Mergoum (2007) report four QTL across chromosomes 2B, 3B, and 7A (two loci) associated with high kernel shattering in a Sumai3 derived population. Moreover, Jantasuriyarat et al (2004) report six QTL that affected threshability on chromosome arms 2AS, 2BL, 2DS, 5AL, 6AS and 6DL in recombinant inbred lines of the International Triticeae Mapping Initiative (ITMI) population, W-7984/Opata 85.

Although no report was found on epistatic interactions of shattering resistance QTL in wheat, epistatic interactions for other traits have been reported such as resistance to wheat rust (Singh et al. 2013, 2014) and common bunt (Bokore et al. 2019; Singh et al. 2016). As epistasis describes genetic interactions in terms of how phenotypic effects of an allele depend on another allele in the genome (Chou et al. 2011), the understanding of such epistatic interactions provides additional information on the most desirable allele combinations (Cheverud and Routman 1995; Singh et al. 2013). Knowledge of epistasis is helpful to understand how certain genes may function synergistically, and the contribution of epistasis to additive genetic or breeding value of interacting genes.

Most of the wheat spike shattering response studies (Jantasuriyarat et al. 2004; Marza et al. 2006; Zhang and Mergoum 2007) are based on simple sequence repeat (SSR) markers. Advancement in next-generation sequencing (NGS) technologies has contributed toward high throughput discovery of large numbers of single nucleotide polymorphisms (SNPs) revolutionizing genetic mapping. Also, the shift in DNA marker technologies from fragment-based polymorphism including amplified fragmented length polymorphism (AFLP) and SSR markers to sequence-based SNP markers provides a huge opportunity for constructing high-density genetic maps ultimately resulting in an increase in the number of informative markers. Polymorphisms from differences at a single nucleotide (substitution, deletion or insertion) occur frequently and can be associated with phenotypes (Grover and Sharma 2016). Higher incidence of markers allows better coverage of the genome revealing more trait-related loci with greater resolution. Mapping of many traits has benefitted from SNP mapping, as can be the case for shattering response in wheat for the development of breeder friendly markers.

It is essential to understand the genetic basis of shattering resistance to maintain the balance between threshability and shattering traits through marker-assisted selection. Genes affecting shattering resistance and their epistatic interaction in contemporary Canadian spring wheat cultivars such as Carberry (DePauw et al. 2011) and AC Cadillac (DePauw et al. 1998) have not been characterized. They derive from different genealogical lineages and under very windy conditions after reaching maturity, shattering has occurred (personal observations by DePauw). The objectives of this study were to determine and map genomic regions controlling spike shattering in Canada Western Red Spring wheat cultivars Carberry and AC Cadillac, to investigate epistatic interactions among these loci and to determine the relationship of the shattering trait with plant height.

## Materials and methods

### Phenotyping

Two doubled haploid (DH) populations Carberry/AC Cadillac (775 lines) and Carberry/Thatcher (297 lines) were evaluated for spike shattering near Swift Current, SK., in a series of nurseries of differing environmental conditions. Carberry is a semi-dwarf doubled haploid, hard red spring wheat cultivar that derives from the cross Alsen and Superb made in 2000 at the Swift Current Research and Development Centre, AAFC, SK, Canada and registered in 2009. AC Cadillac is a hard red spring wheat adapted to the Canadian Prairies with shattering resistance similar to Katepwa. Thatcher was selected from a double cross Marquis/ Iumillo// Marquis/ Kanred wheat in 1925 and released in 1935 (Hayes et al. 1936). In studies of other traits, segregation for shattering was observed in the two populations.

The Carberry/AC Cadillac population was planted in 3-m-long single row nurseries in 2012 and 2013 at a site named South Farm and in 1.5-m rows in 2012 and 2013 at a nursery site named Centre Farm and in 2014 at Field 16 North Farm of the Swift Current Research and Development Centre. The Carberry/Thatcher population was evaluated in 1.5-m-long single rows in Centre Farm nurseries near Swift Current in 2016 and 2018, and 3-m-long single rows in 2018 at Field 17 North Farm and in 2019 at South Farm. South Farm has coordinates lat. 50°16' N., long. 107°44' W. The South Farm has a Swinton loam soil (Orthic Brown Chernozem) at 825 m above sea level, while the Centre Farm has a slightly higher clay content, and the North Farm Field 16 and 17 is a heavy clay soil at about 750 m asl. All nurseries were irrigated except South Farm, 2012, 2013 and 2019. The lines were inoculated with common bunt [*Tilletia laevis* Kühn in Rabenh., and *T. tritici* (Bjerk.) G. Wint. in Rabenh.] races L16 and T19 (Hoffmann and Metzger 1976) in the nurseries (except the 3 m rows in 2018), but the populations expressed a high level of resistance to bunt such that the effect on shattering was minimal. The shattering score was based on the proportion of the spike with loss of kernels x the proportion of the spikes within a plot with symptoms of seed shattering × 100 where 1 was no shattering, 9 was all spikes with a bare rachis and the rest of the numbers fell in between compressed at the lower tail.

Pearson’s correlation analysis was performed on the shattering data using SAS (SAS Institute, Cary, NC) to determine the repeatability of the shattering scores across environments in each of the two mapping populations. Also, correlation analysis was performed for the shattering response with plant height to assess the relationship between these two traits. Broad sense heritability of the spike shattering expressed as the ratio of the genetic variance and the phenotypic variance was determined.

### Genotyping, construction of linkage maps and QTL analysis

The DNA of parents and DH lines was extracted from the first leaf of seedlings with the BioSprint 96 DNA Plant Kit (QIAGEN Science, Maryland, USA). The parents and 297 DH lines of the Carberry/Thatcher and 775 lines of the Carberry/AC Cadillac populations were genotyped with the 90 K Infinium iSelect SNP wheat assay (Illumina Inc., San Diego, CA). Linkage maps of the two populations were built using the two-step mapping strategy as previously described (Bokore et al. 2019; Fowler et al. 2016). Main effect QTL associated with shattering resistance were identified by performing QTL analysis with MapQTL.6 ® (Van Ooijen 2009). The permutation test option (1000 permutations) within MapQTL was applied to determine the significance threshold for the logarithm of the odds (LOD). Genome-wide threshold levels were used to declare significant QTL at the 5% level of significance. Automatic co-factor detection based on backward elimination to identify the co-factor markers as well as manual co-factor selection was performed for Multiple QTL Mapping (MQM). The data were square root and logarithm transformed and QTL analyses were repeated.

### Epistasis analysis

Additive x additive epistasis between the main effect QTL was performed using QTLNetwork 2.0 (Yang et al. 2008, 2007). The additive epistatic interactions were estimated using the “map epistasis” option of QTLNetwork. QTL with individual or epistatic effects were determined using the “two-dimensional (2D) genome scan” option. The 2D genome scan option enables mapping epistatic QTL with or without single-locus effects. The critical *F* values were determined with 1000 permutations.

## Results

### Response of the genotypes to shattering

**Fig. 1:**
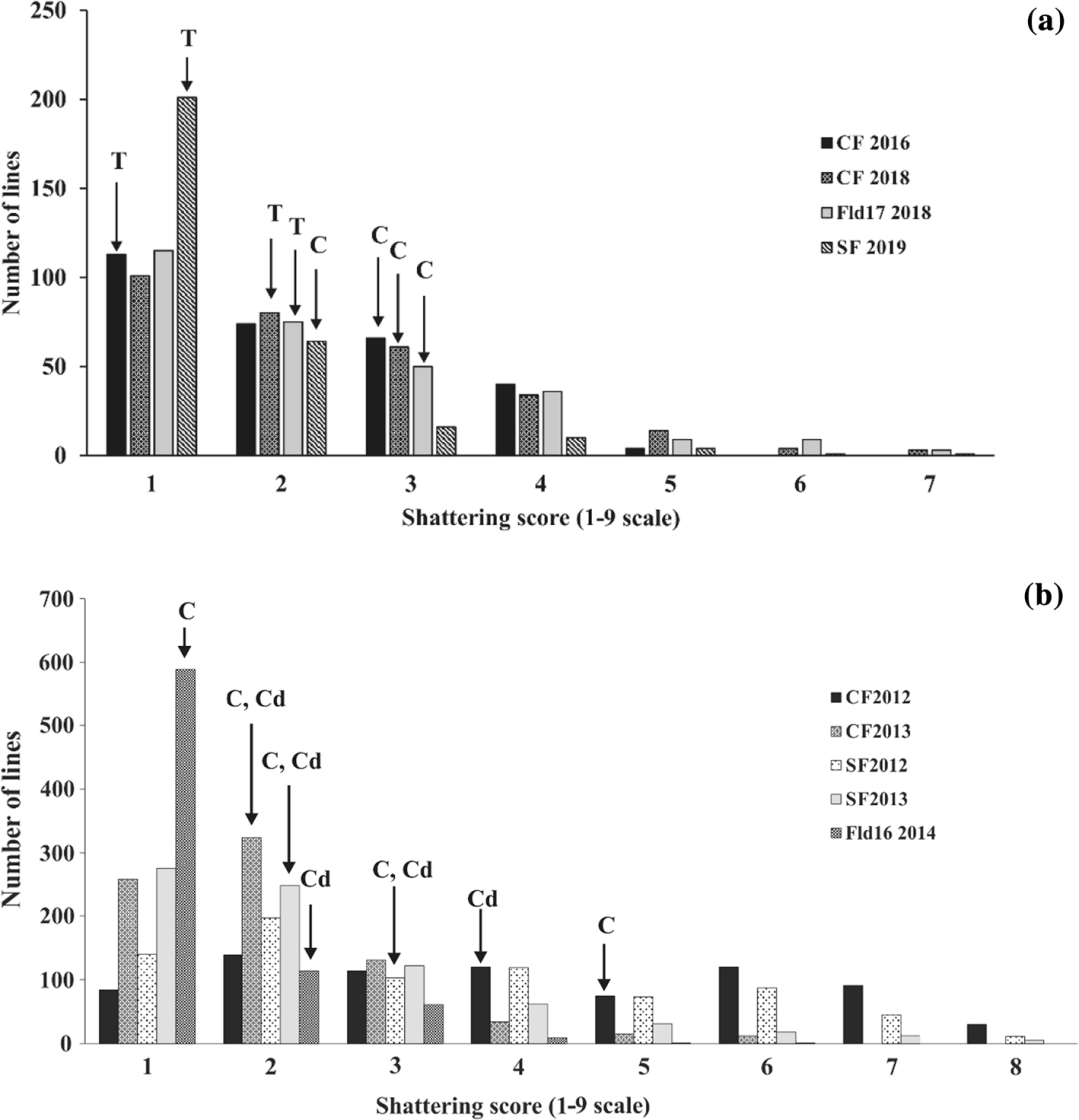
Phenotypic distribution for shattering resistance in the (**a**) Carberry/Thatcher and (**b**) Carberry/AC Cadillac doubled haploid populations evaluated near Swift Current, SK in different years. Abbreviations for location names followed by years of field experimentation are as follows: CF, Centre Farm; SF, South Farm; Fld16, Field 16; and Fld17, Field 17. Shattering scores of the parents Carberry (**c**), AC Cadillac (Cd) and Thatcher (T) are indicated by dark arrows

Thatcher consistently expressed lower shattering, scores ranging from 1.0 to 1.8 with a mean over environments of 1.5, than Carberry, ranging from 1.8 to 2.8 with a mean of 2.3 (Table 1). Thatcher had significantly lower shattering scores than Carberry in three of four environments. Whereas Carberry and AC Cadillac scores were more comparable with equal means over environments of 2.4. Both Carberry’s shattering scores (range: 1.1–4.9) and AC Cadillac’s (range: 1.6–3.5) were wider ranging in the Carberry/AC Cadillac experiments than the Carberry/Thatcher experiments. However, Carberry’s mean score across environments was similar at 2.3 in the Carberry/Thatcher experiments and 2.4 in the Carberry/AC Cadillac experiments. The phenotypic distributions of the lines were typically continuous and skewed to the right with a preponderance of low shattering types (Fig. 1a and b). The populations displayed a wide range of variation in expression from 1 to 7 for Carberry/Thatcher and 1 to 8 for Carberry/AC Cadillac. Transgressive segregation was observed in the populations particularly with the segregation of high shattering lines. The square root and log transformations of the data resulted in similar patterns to the untransformed data are presented.

**Table 1:** Mean, range, standard error (SE) and variance components generated by epistasis analysis using QTLNetwork software on mean of spike shattering scores (1–9 scale) in the Carberry/Thatcher and Carberry/Cadillac populations evaluated in different environments

Location-year	Shattering score (1–9 scale) ^a^					Variance components ^b^			
	Range	Mean	SE	Thatcher	Carberry	V(G)/V(P)	V(E)/V(P)	V(A)/V(P)	V(AA)/V(P)
*Carberry/Thatcher*									
Centre Farm 2016	1–5	2.2	0.06	1.4	2.7	0.35	0.65	0.31	0.04
Centre Farm 2018	1–7	2.3	0.08	1.8	1.9	0.42	0.58	0.34	0.08
Field 17 2018	1–7	2.3	0.08	1.8	2.8	0.39	0.61	0.36	0.03
South Farm 2019	1–7	1.5	0.05	1.0	1.8	0.33	0.67	0.21	0.11

Carberry/AC Cadillac				AC Cadillac	Carberry				
Centre Farm 2012	1–8	4.1	0.07	3.5	4.9	0.59	0.41	0.58	0.01
Centre Farm 2013	1–6	2.0	0.04	2.3	2.1	0.39	0.61	0.36	0.03
South Farm 2012	1–8	3.4	0.07	2.9	2.5	0.64	0.36	0.61	0.03
South Farm 2013	1–8	2.3	0.05	1.9	1.5	0.54	0.46	0.48	0.07
Field 16 2014	1–6	1.4	0.03	1.6	1.1	0.22	0.78	0.20	0.02

^a^The score was based on the proportion of the spike with loss of kernels x the proportion of the spikes within a plot with symptoms of seed shattering × 100 where 1 was no shattering, 9 was all spikes had a bare rachis, and the rest of the numbers fell in between compressed at the lower tail

^b^V(G), genotype variance; V(A), additive variance; AA, additive x additive variance; V(P), phenotypic variance, V(E), environmental variance V(G)/V(P), broad sense heritability; V(A)/V(P), narrow sense heritability; (V(AA)/V(P), additive x additive epistasis heritability; V(E)/V(P), environmental effect

Variance components and heritability estimates of the shattering trait are presented in Table 1. Estimates of broad sense heritability based on the ratio of the genetic variance to the total variance among the scores of the lines ranged from 0.22 to 0.64 for Carberry/AC Cadillac population while for the Carberry/Thatcher population it ranged from 0.33 to 0.42. Narrow sense heritabilities were somewhat lower than the broad sense heritabilities. The heritability due to the additive epistatic interactions was much lower than both broad sense and narrow sense heritabilities.

Correlation matrix analysis between shattering determinations and plant height for both populations (Table 2) detected highly significant (*P* < 0.001) positive correlations within both plant height and shattering location-year combinations as well as between trait location-year combinations. The correlations among shattering scores between environments were moderate to high, ranging from 0.46 to 0.80 for the Carberry/AC Cadillac population, and from 0.56 to 0.76 for Carberry/Thatcher. Relative to other test environments, the correlations involving Field 16 in 2014 tended to be lower. The correlations between plant height scores were high ranging from 0.83 to 0.89 for the Carberry/AC Cadillac population and were 0.88 for Carberry/Thatcher. Highly significant (*P* < *0.001*) positive inter-trait correlations of shattering and plant height were low-to-moderate ranging from 0.15 to 0.41 in Carberry/AC Cadillac and 0.26 to 0.48 in the Carberry/Thatcher population.

**Table 2:** Pearson’s correlation between locations and years for shattering and plant height of the Carberry/Thatcher and Carberry/AC Cadillac populations evaluated near Swift Current, SK

Carberry/AC Cadillac	SH South Farm 2012	SH South Farm 2013	SH Centre Farm 2013	SH Field 16 SC2014	PHT Centre Farm 2011	PHT Centre Farm 2012	PHT Centre Farm 2013	PHT South Farm 2013
SH Centre Farm 2012	0.80^a^	0.71	0.70	0.48	0.26	0.30	0.29	0.31
SH South Farm 2012	–	0.77	0.67	0.47	0.37	0.41	0.41	0.43
SH South Farm 2013		–	0.74	0.53	0.40	0.43	0.46	0.49
SH Centre Farm 2013			–	0.47	0.31	0.33	0.35	0.36
SH Field 16 SC2014				–	0.15	0.16	0.16	0.19
PHT Centre Farm 2011					–	0.84	0.86	0.83
PHT Centre Farm 2012						–	0.88	0.87
PHT Centre Farm 2013							–	0.89

Carberry/Thatcher	SH Centre Farm 2018	SH Field 17 2018	SH South Farm 2019	PHT Centre Farm 2014	PHT Centre Farm 2015	PHT Centre Farm 2016
SH Centre Farm 2016	0.68	0.68	0.56	0.29	0.27	0.28
SH Centre Farm 2018	–	0.76	0.66	0.42	0.40	0.41
SH Field 17 2018		–	0.70	0.48	0.48	0.47
SH South Farm 2019			–	0.30	0.29	0.26
PHT Centre Farm 2014				–	0.88	0.88
PHT Centre Farm 2015					–	0.88

^a^SH, shattering response; PHT, plant height

^b^All correlation values are significant at *P* < 0.0001

### Construction of linkage maps

The summary statics of high-density SNP linkage maps of the Carberry/Thatcher and Carberry/AC Cadillac populations are presented in Supplemental Table 1. For the Carberry/Thatcher population, a total of 8360 polymorphic markers were mapped on 28 linkage groups corresponding to 20 wheat chromosomes, covering 3645.8 cM of the wheat genome with an average density of 0.6 cM per marker. All except chromosome 4D were represented in the map. The minor allele frequency (MAF) ranged from 0.37 to 0.50 with an average of 0.47, whereas the polymorphism information content (PIC) ranged from 0.46 to 0.50 with an average of 0.50. The Carberry/AC Cadillac population map involved a total of 6806 SNP markers mapped on 29 linkage groups, covering 3237.9 cM of the wheat genome and an average density of 0.72 cM per marker. The MAF for the Carberry/AC Cadillac population ranged from 0.41 to 0.50 with an average of 0.48, and the PIC ranging from 0.48 to 0.50 with an average of 0.48.

### QTL identified in the Carberry/Thatcher population

**Fig. 2:**
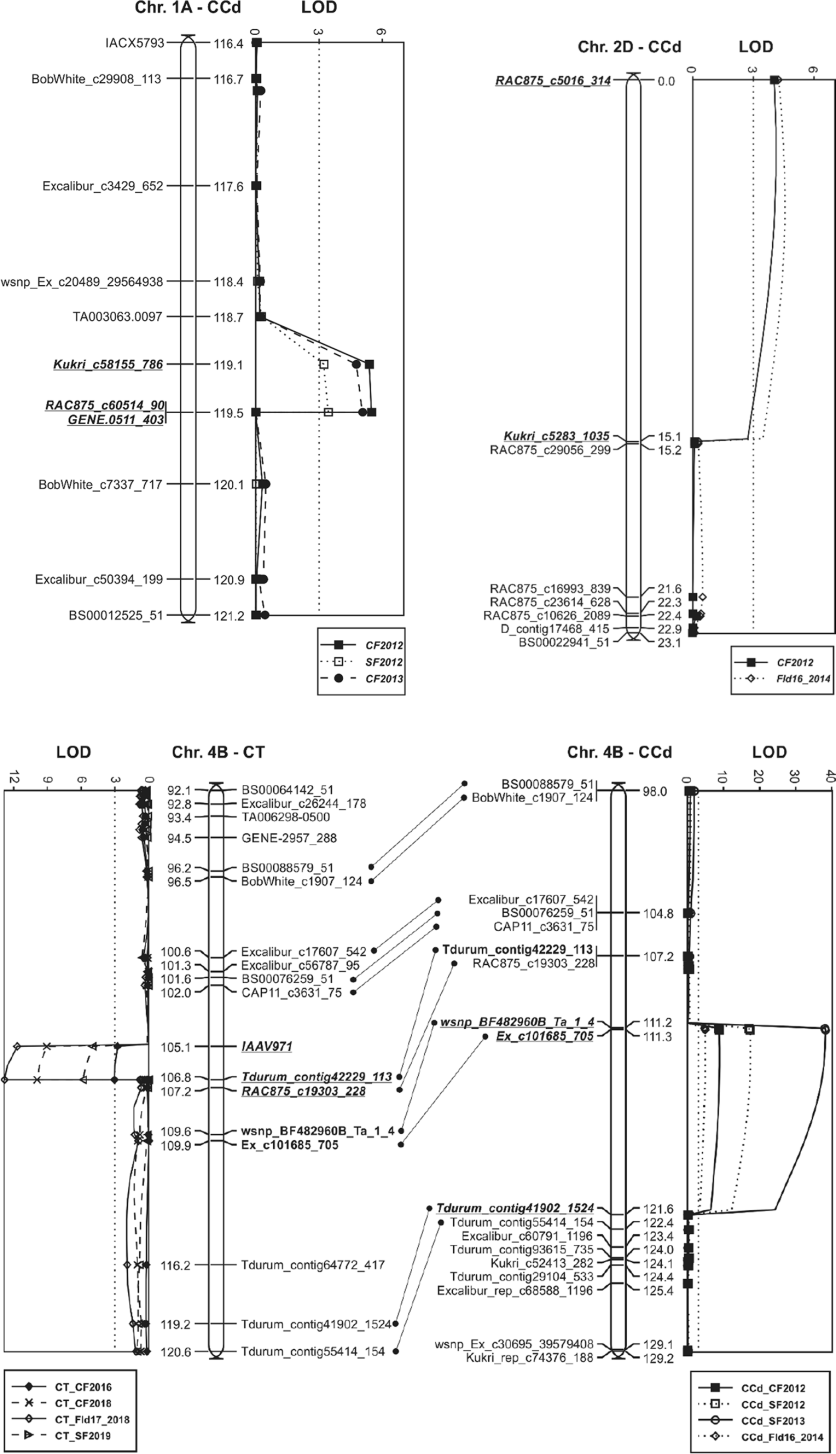

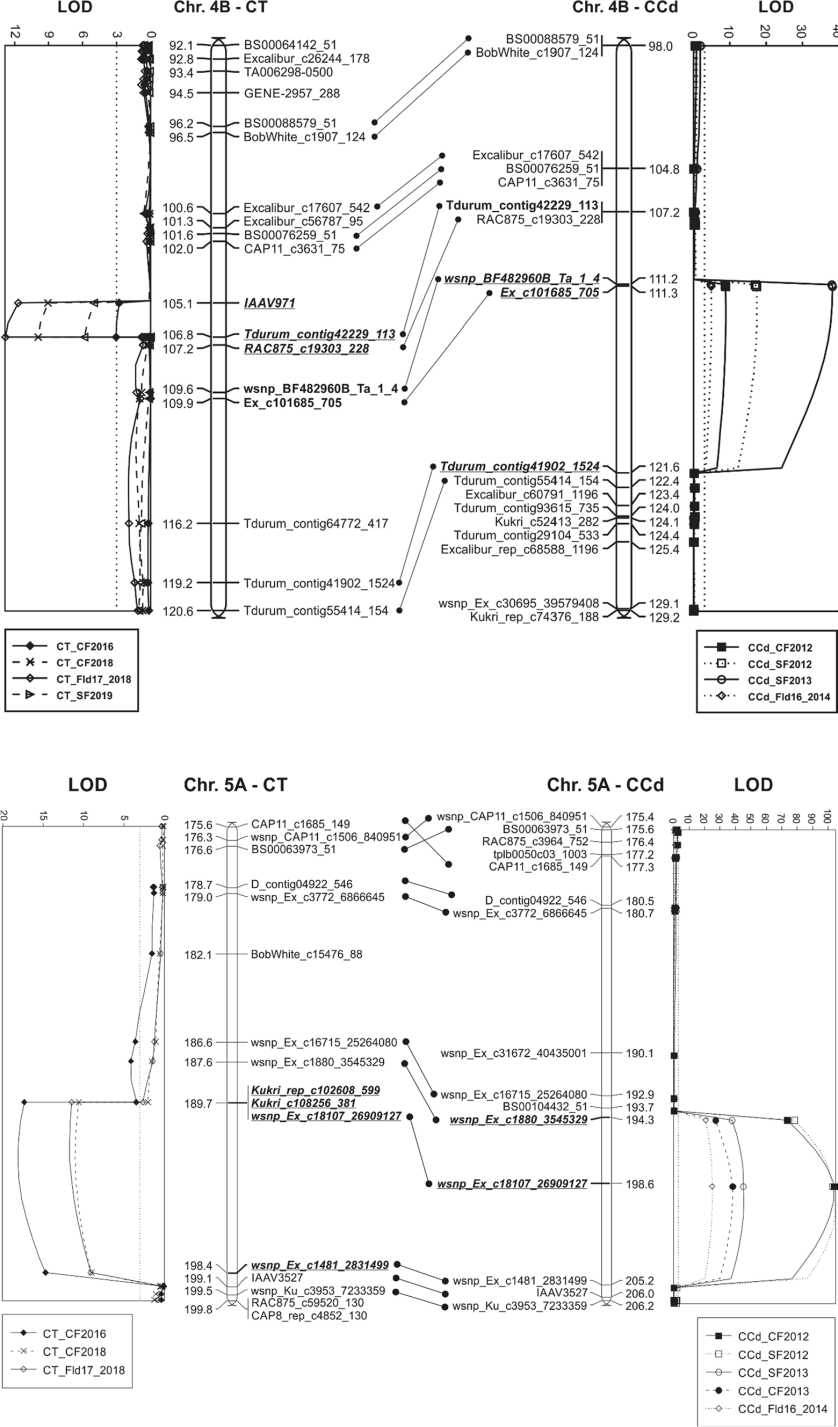

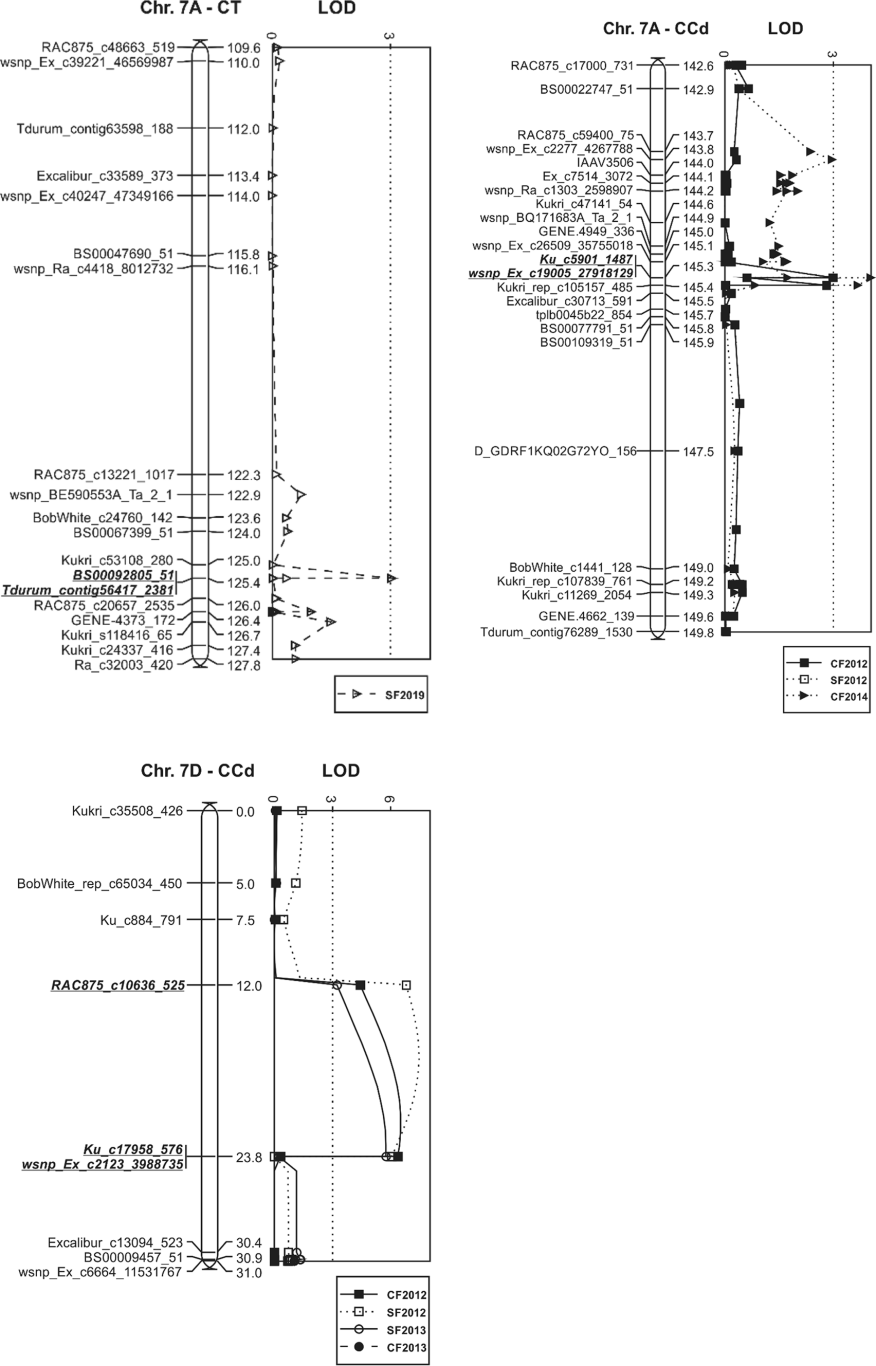
Linkage maps displaying shattering resistance QTL identified in the Carberry/Thatcher (CT) and Carberry/AC Cadillac (CCd) doubled haploid populations. Logarithm of the odds (LOD) values generated by Multiple QTL Mapping (MQM) analysis are presented alongside linkage maps indicating distances in cM between the 90 K SNP wheat iSelect markers (Illumina Inc., San Diego, CA). Co-segregating markers outside of the QTL intervals were removed from the map. Alleles for reduced shattering on chromosomes 4B, 5A and 7A were derived from Carberry and 1A, 2D, 3A, 3D and 7D from AC Cadillac. The map positions of the QTL on 4B and 5A were aligned across both populations. Abbreviations for locations followed by test year are defined as follows: CF, Centre Farm; SF, South Farm; and field name followed by test year: Fld16_2014, Field 16 2014; and Fld17_2018, Field 17 2018

A summary of QTL associated peak markers, map position (cM), LOD score, phenotypic variation explained (PVE) and additive effects of shattering response loci identified in Carberry/Thatcher and Carberry/AC Cadillac is presented in Table 3. Figure 2 displays linkage maps of the QTL identified in the population. The analysis by MapQTL detected two consistent shattering resistance QTL, one located on chromosomes 4B (designated as *Sh.Sparc-4B*) and the other on 5A (*Sh.Sparc-5A),* along with one sporadic QTL on 7A (*Sh.Sparc-7A.1*). Carberry was the contributor of the low shattering alleles for the three loci, whereas no QTL was contributed by Thatcher. The 4B and 5A loci appeared in all four test environments, but the 7A locus was marginally significant with a LOD score of 3.0 and identified in one environment only. The QTL interval of *Sh.Sparc-5A* is quite broad with several markers present, whereas *Sh.Sparc-4B* is narrow with few associated markers (Fig. 2).

**Table 3:** Closest marker to QTL based on peak LOD, marker position (cM) in the genetic map of respective populations, LOD score, phenotypic variations explained (PVE), mean phenotypic value associated with the parent contributing the allele, and additive value for spike shattering resistance QTL and plant height QTL identified in different environments in Carberry/Thatcher and Carberry/AC Cadillac populations. The analysis was performed by the multiple QTL mapping (MQM) option of MapQTL 6

Environment	Trait name	QTL	Peak marker	Position, cM	LOD score^a^	Phenotypic mean of allele for parent		PVE^b^, %	Additive effect^c^
Carberry/Thatcher						Thatcher	Carberry		
Center Farm 2016	Shattering	*Sh.Sparc-4B*	*Tdurum_contig42229_113*	106.8	3.1	2.4	1.9	4.6	0.2
Center Farm 2018	Shattering	*Sh.Sparc-4B*	*Tdurum_contig42229_113*	106.8	9.9	2.9	1.9	14.2	0.5
Field 17 2018	Shattering	*Sh.Sparc-4B*	*Tdurum_contig42229_113*	106.8	12.8	2.9	1.7	18.0	0.6
South Farm 2019	Shattering	*Sh.Sparc-4B*	*Tdurum_contig42229_113*	106.8	5.8	1.7	1.2	8.7	0.3
Centre Farm 2014	Plant height	*Pht.Sparc-4B*	*Tdurum_contig42229_113*	106.8	55.9	101.5	83.4	58.0	9.1
Centre Farm 2015	Plant height	*Pht.Sparc-4B*	*Tdurum_contig42229_113*	106.8	51.3	88.6	75.2	54.9	6.7
Centre Farm 2016	Plant height	*Pht.Sparc-4B*	*Tdurum_contig42229_113*	106.8	58.6	106.5	89.0	59.7	8.8
Centre Farm 2016	Shattering	*Sh.Sparc-5A*	*Kukri_rep_c102608_599*	189.67	18.1	2.7	1.5	24.5	0.6
Centre Farm 2018	Shattering	*Sh.Sparc-5A*	*Kukri_rep_c102608_599*	189.67	11.0	2.9	1.8	15.7	0.6
Field 17 2018	Shattering	*Sh.Sparc-5A*	*Kukri_rep_c102608_599*	189.67	11.7	2.9	1.7	16.6	0.6
South Farm 2019	Shattering	*Sh.Sparc-5A*	*Kukri_rep_c102608_599*	189.67	7.3	1.8	1.1	10.6	0.3
South Farm 2019	Shattering	*Sh.Sparc-7A.1*	*BS00092805_51 / Tdurum_contig56417_2381*	125.4	3.0	1.7	1.3	4.6	0.2

Carberry/AC Cadillac						AC Cadillac	Carberry		
Centre Farm 2012	Shattering	*Sh.Sparc-1A*	*RAC875_c60514_90*	119.46	5.5	3.7	4.5	3.2	− 0.4
South Farm 2012	Shattering	*Sh.Sparc-1A*	*RAC875_c60514_90*	119.46	3.4	3.1	3.7	2.0	− 0.3
South Farm 2013	Shattering	*Sh.Sparc-1A*	*RAC875_c60514_90*	119.46	2.8	2.1	2.5	1.6	− 0.2
Centre Farm 2013	Shattering	*Sh.Sparc-1A*	*RAC875_c60514_90*	119.46	5.1	1.9	2.2	3.0	− 0.2
Centre Farm 2012	Shattering	*Sh.Sparc-2D*	*RAC875_c5016_314*	0	4.0	3.8	4.4	2.4	− 0.3
South Farm 2012	Shattering	*Sh.Sparc-2D*	*RAC875_c5016_314*	0	2.4	3.2	3.6	1.4	− 0.2
Field 16 2014	Shattering	*Sh.Sparc-2D*	*RAC875_c5016_314*	0	4.3	1.3	1.5	2.5	− 0.1
Centre Farm 2012	Shattering	*Sh.Sparc-3A*	*Wsnp_Ku_C44716_51926415*	101.45	3.1	3.8	4.4	1.8	− 0.3
Centre Farm 2013	Shattering	*Sh.Sparc-3A*	*Wsnp_Ku_C44716_51926415*	101.45	3.3	1.9	2.2	1.9	− 0.1
Centre Farm 2012	Shattering	*Sh.Sparc-3D*	*wsnp_Ex_c1032_1972537*	11.87	4.6	3.8	4.4	2.7	− 0.3
Centre Farm 2012	Shattering	*Sh.Sparc-4B*	*wsnp_BF482960B_Ta_1_4*	111.2	8.8	4.6	3.6	5.1	0.5
South Farm 2012	Shattering	*Sh.Sparc-4B*	*wsnp_BF482960B_Ta_1_4*	111.2	17.5	4.0	2.8	9.8	0.6
Centre Farm 20,113	Shattering	*Sh.Sparc-4B*	*wsnp_BF482960B_Ta_1_4*	111.2	17.9	2.4	1.7	10.1	0.3
South Farm 20,113	Shattering	*Sh.Sparc-4B*	*wsnp_BF482960B_Ta_1_4*	111.2	38.3	3.0	1.7	20.3	0.7
Field 16 2014	Shattering	*Sh.Sparc-4B*	*Ex_c101685_705*	111.33	4.8	1.5	1.2	2.8	0.1
Centre Farm 2011	Plant height	*Pht.Sparc-4B*	*wsnp_BF482960B_Ta_1_4*	111.2	123.0	98.3	84.8	51.9	6.8
Centre Farm 2012	Plant height	*Pht.Sparc-4B*	*wsnp_BF482960B_Ta_1_4*	111.2	116.0	99.1	86.3	49.8	6.4
Centre Farm 2013	Plant height	*Pht.Sparc-4B*	*wsnp_BF482960B_Ta_1_4*	111.2	146.5	110.4	93.1	58.1	8.6
South Farm 2013	Plant height	*Pht.Sparc-4B*	*wsnp_BF482960B_Ta_1_4*	111.2	183.2	116.5	94.9	66.3	10.8
Centre Farm 2012	Shattering	*Sh.Sparc-5A*	*wsnp_Ex_c18107_26909127*	198.63	103.7	5.4	2.6	46.0	1.4
South Farm 2012	Shattering	*Sh.Sparc-5A*	*wsnp_Ex_c18107_26909127*	198.63	102.6	4.6	2.0	46.2	1.3
Centre Farm 2013	Shattering	*Sh.Sparc-5A*	*wsnp_Ex_c18107_26909127*	198.63	38.2	2.5	1.5	20.3	0.5
South Farm 2013	Shattering	*Sh.Sparc-5A*	*wsnp_Ex_c18107_26909127*	198.63	45.0	3.0	1.5	23.6	0.7
Field 16 2014	Shattering	*Sh.Sparc-5A*	*wsnp_Ex_c18107_26909127*	198.63	24.9	1.6	1.1	13.8	0.3
Centre Farm 2011	Plant height	*Pht.Sparc-5A*	*BS00077855_51*	139.6	4.2	92.5	89.5	2.5	1.5
Centre Farm 2012	Plant height	*Pht.Sparc-5A*	*BS00077855_51*	139.6	6.4	93.9	90.4	3.7	1.8
Centre Farm 2013	Plant height	*Pht.Sparc-5A*	*BS00077855_51*	139.6	6.2	103.2	98.9	3.6	2.2
South Farm 2013	Plant height	*Pht.Sparc-5A*	*BS00077855_51*	139.6	5.7	107.2	102.4	3.3	2.4
Centre Farm 2012	Shattering	*Sh.Sparc-7A.2*	*wsnp_Ex_c19005_27918129*	145.27	3.0	4.4	3.8	1.8	0.3
South Farm 2012	Shattering	*Sh.Sparc-7A.2*	*wsnp_Ex_c19005_27918129*	145.27	4.1	3.7	3.1	2.4	0.3
Field 16 2014	Shattering	*Sh.Sparc-7A.2*	*wsnp_Ex_c19005_27918129*	145.27	4.0	1.5	1.2	2.4	0.1
Centre Farm 2012	Shattering	*Sh.Sparc-7D*	*Ku_c17958_576*	23.78	6.5	3.7	4.6	3.7	− 0.4
South Farm 2012	Shattering	*Sh.Sparc-7D*	*RAC875_c10636_525*	12.0	7.5	3.0	3.8	3.9	− 0.4
South Farm 2013	Shattering	*Sh.Sparc-7D*	*Ku_c17958_576*	23.78	5.8	2.0	2.6	3.4	− 0.3
Centre Farm 2013	Shattering	*Sh.Sparc-7D*	*Ku_c17958_576*	23.78	5.8	1.8	2.3	3.4	− 0.2

^a^Maximum likelihood LOD score for the QTL

^b^Phenotypic variation explained by the QTL

^c^Positive additive effect indicates an increasing value of the trait from Thatcher and AAC Cadillac; negative additive effect indicates an increasing value of the trait from Carberry

*Sh.Sparc-5A* was most highly associated with SNP markers *Kukri_rep_c102608_599* and *wsnp_Ex_c1481_2831499* (Fig. 2) located on chromosome arm 5AL (Wang et al. 2014). This QTL had a LOD score as high as 18.1 (Table 3). *Sh.Sparc-4B* was associated with *IAAV971* and *Tdurum_contig42229_113* (Fig. 2 and Table 3) on chromosome arm 4BS (Wang et al. 2014)*. Sh.Sparc-7A.1* was associated with markers *BS00092805_51* and *Tdurum_contig56417_2381* located on 7AS (Table 3 and Fig. 2). The phenotypic variation explained by the 4B locus, ranging from 4.6 to 18.0%, was somewhat less than for the 5A ranging from 10.6 to 24.5%. The 4B and 5A loci cumulatively explained 29.2% of the total phenotypic variation at Centre Farm in 2016, 29.9% of the variation at Centre Farm in 2018, 34.6% at Field 17 in 2018 and 19.3% at South Farm in 2019.

### QTL identified in the Carberry/AC Cadillac population

A total of eight main effect QTL for shattering resistance were detected on chromosomes 1A (designated *Sh.Sparc-1A*), 2D (*Sh.Sparc-2D*), 3A (*Sh.Sparc-3A*), 3D (*Sh.Sparc-3D*), 4B (*Sh.Sparc-4B*), 5A (*Sh.Sparc-5A*), 7A (*Sh.Sparc-7A.2*) and 7D (*Sh.Sparc-7D*) in the Carberry/AC Cadillac population (Table 3). Three of the QTL, on 4B, 5A and 7A, were derived from Carberry, and those QTL on 1A, 2D, 3A, 3D and 7D were contributed by AC Cadillac. The QTL on 4B and 5A were detected in all the five test environments, the QTL on 1A and 7D in four out of five, 2D and 7A in three out of five, 3A in two of five, and 3D in a single environment.

The Carberry/AC Cadillac QTL on 4B and 5A mapped in the same chromosomal region as the Carberry/Thatcher population (Fig. 2) and are designated with the same names. The *Sh.Sparc-5A* was associated with SNP markers *wsnp_Ex_c1880_3545329* and *wsnp_Ex_c18107_26909127* located on 5AL (Wang et al. 2014). The QTL, *Sh.Sparc-5A,* had the highest LOD value (103.7) of any QTL and explained the most phenotypic variation ranging from 13.8 to 46.2% across environments. The *Sh.Sparc-4B* associated with *wsnp_BF482960B_Ta_1_4* and *Tdurum_Contig41902_1524* was located on 4BS. The QTL had a maximum LOD value of 38.3 with the phenotypic variation explained ranging from 5.2 to 20.3%. In 2012, the 4B and 5A loci alone explained 51.1% of the phenotypic variation at Centre Farm and 56.0% at South Farm. In 2013 both 4B and 5A explained 30.4% at Centre Farm and 43.9% at South Farm, and in 2014 the two explained 16.6% of the variation at Field 16. The third locus from Carberry, *Sh.Sparc-7A.2,* explained phenotypic variation ranging from 1.8 to 2.4%. The SNP markers associated with the 7A locus in Carberry/AC Cadillac, *wsnp_Ex_c19005_27918129* and *Kukri_rep_c105157_485,* mapped to the long arm of the chromosome (Wang et al. 2014) and were located about 448 Mb from *Sh.Sparc-7A.1* in the bread wheat reference genome sequence (RefSeq v2.0).

The QTL derived from AC Cadillac tended to explain a smaller proportion of the phenotypic variation than Carberry, with the AC Cadillac variation ranging across environments from 2.4 to 5.5% for *Sh.Sparc-1A*, 1.4 to 2.5% for *Sh.Sparc-2D*, 1.5 to 1.9% for *Sh.Sparc-3A* and explaining 2.7% for *Sh.Sparc-3D. Sh.Sparc-7D* explained the greatest variation from AC Cadillac ranging from 3.4 to 3.9%. Considering chromosomal locations of AC Cadillac derived QTL, associated markers for the *Sh.Sparc-1A* were assigned to chromosome arm 1AL (Wang et al. 2014). Similarly markers for *Sh.Sparc-2D* were assigned to 2DL, *Sh.Sparc-3A* markers to 3AL, *Sh.Sparc-3D markers* to 3DL and *Sh.Sparc-7D* markers to 7DS (Wang et al. 2014).

The 4BS QTL Carberry alleles for reduced spike shattering are in coupling with reduced plant height (designated *Pht.Sparc-4B*) in both Carberry/Thatcher and Carberry/AC Cadillac populations (Table 3). The 5AL QTL alleles for reduced plant height (*Pht.Sparc-5AL*) within the Carberry/AC Cadillac population were contributed by Carberry as was the 5AL shattering resistance QTL, but the plant height QTL was not segregating in the Carberry/Thatcher population.

### Epistatic interaction

Apart from epistatic interactions, QTLNetwork detected the same main effect QTL as MapQTL except for the 7AS QTL that was identified by MapQTL at a single environment in Carberry/Thatcher. The analysis revealed nine significant digenic interactions (Table 4). A1 x A2 epistatic interaction effects with positive additive values contributed to reduced shattering responses while those with negative values contributed to increased shattering responses. Only *Sh.Sparc-4B/5A* was significant in the Carberry/Thatcher population whereas all nine digenic interactions were significant in Carberry/AC Cadillac. The Centre Farm 2012 environment revealed only one significant epistatic interaction (*Sh.Sparc-3A/Sh.Sparc-4B*) compared with other environments for which at least two significant interactions were detected. Two of the nine pairs of epistatic interactions contributed to reduced shattering scores (A1 x A2 effect is positive), whereas the remaining seven pairs represented antagonistic interactions (Table 4). The first positive epistatic interaction was between *Sh.Sparc-4B* and *Sh.Sparc-5A* observed in both populations across multiple environments. The second positive interaction was between *Sh.Sparc*-*5A* and *Sh.Sparc*-*7A.2,* which occurred in the single environment Field 16, 2014 in the Carberry/AC Cadillac population. The Field 16 2014 test was characterized by the lowest Carberry/AC Cadillac shattering scores.

**Table 4:** Additive x additive (A1 x A2) epistatic interactions detected by QTLNetwork for spike shattering scores (1–9 scale), interacting QTL intervals (QTL1 and QTL2), and levels of significance (*P* value) for QTL identified in the Carberry/Thatcher and Carberry/AC Cadillac doubled haploid populations evaluated near Swift Current, Canada in different years

	QTL1	Interval 1	Position, cM	QTL2	Interval 2	Position, cM	A1 x A2 effect	*P* Value
Carberry/Thatcher								
Centre Farm 2016	4B	*BS00081631_51-Tdurum_Contig55414_154*	96.16–120.58	5A	*Wsnp_Ex_C1880_3545329 - Wsnp_Ex_C18107_26909127*	187.62–189.67	0.25	< 0.001
Centre Farm 2018	4B	*IACX557-Tdurum_Contig64772_417*	109.91–121.5	5A	*Wsnp_Ex_C18107_26909127 - Wsnp_Ex_C1481_2831499*	189.67–198.41	0.39	< 0.001
Field 17 2018	4B	*IACX557-Tdurum_Contig64772_417*	109.91–116.24	5A	*Wsnp_Ex_C16715_25264080 - Wsnp_Ex_C1880_3545329*	186.59–187.62	0.25	< 0.001
South Farm 2019	4B	*IACX557-Tdurum_Contig64772_417*	109.91–116.24	5A	*Wsnp_Ex_C18107_26909127 - Wsnp_Ex_C1481_2831499*	189.67–198.41	0.33	< 0.001
Location-year mean	4B	*IACX557-Tdurum_Contig64772_417*	109.91–116.24	5A	*Wsnp_Ex_C16715_25264080 - Wsnp_Ex_C1880_3545329*	186.59– 187.62	0.28	< 0.001

Carberry/AC Cadillac								
Centre Farm 2012	3A	*Wsnp_Ku_C44716_51926415-Wsnp_Rfl_Contig2699_2402527*	101.45–104.65	4B	*Bs00021984_51-Wsnp_Bf482960b_Ta_1_4*	107.87–111.2	− 0.16	< 0.001
South Farm 2012	4B	*Ex_C101685_705 - Tdurum_Contig41902_1524*	111.33–121.56	5A	*Wsnp_Ex_C3772_6866645 - Wsnp_Ex_C31672_40435001*	180.73–190.13	0.13	< 0.005
	5A	*Wsnp_Ex_C3772_6866645 - Wsnp_Ex_C31672_40435001*	180.73–190.13	7D	*Rac875_C10636_525 - Ku_C17958_576*	12–23.78	− 0.18	< 0.001
	7A.2	*Bs00071425_51 - Bs00063458_51*	152.99–156.82	7D	*Rac875_C10636_525 - Ku_C17958_576*	12–23.78	− 0.12	0.006
Centre Farm 2013	1A	*Rac875_C60514_90-Bobwhite_C7337_717*	119.46–120.12	4B	*Ex_C101685_705*–*Tdurum_Contig41902_1524*	111.33–121.56	− 0.07	0.013
	4B	*Excalibur_C29141_864 - Excalibur_C17607_542*	98–104.81	5A	*Wsnp_Ex_C18107_26909127 - Wsnp_Ex_C1481_2831499*	198.63–205.24	0.17	< 0.001
	5A	*Wsnp_Ex_C18107_26909127 - Wsnp_Ex_C1481_2831499*	198.63–205.24	7D	*Ku_C17958_576 - Excalibur_C13094_523*	23.78–30.35	− 0.07	0.015
South Farm 2013	2D	*Rac875_C10626_2089 - Bs00022941_51*	22.39–23.07	5A	*Wsnp_Jd_C43389_30288993 - Cap8_Rep_C4852_130*	206–206.16	− 0.08	0.028
	3A	*Excalibur_C63733_173 - Wsnp_Ku_C44716_51926415*	99.2–101.45	4B	*Bs00021984_51 - Wsnp_Bf482960b_Ta_1_4*	107.87–111.2	− 0.09	0.017
	3A	*Excalibur_C63733_173 - Wsnp_Ku_C44716_51926415*	99.2–101.45	5A	*Wsnp_Ex_C3772_6866645 - Wsnp_Ex_C31672_40435001*	180.73–190.13	− 0.1	0.007
	4B	*Bs00021984_51 - Wsnp_Bf482960b_Ta_1_4*	107.87–111.2	7D	*Ku_C17958_576 - Excalibur_C13094_523*	23.78–30.35	− 0.1	0.006
	4B	*Bs00021984_51 - Wsnp_Bf482960b_Ta_1_4*	107.87–111.2	5A	*Wsnp_Ex_C3772_6866645 - Wsnp_Ex_C31672_40435001*	180.73–190.13	0.2	< 0.001
	5A	*Wsnp_Ex_C3772_6866645 - Wsnp_Ex_C31672_40435001*	180.73–190.13	7D	*Ku_C17958_576 - Excalibur_C13094_523*	23.78–30.35	− 0.16	< 0.001
Field 16 2014	4B	*Excalibur_C29141_864 - Excalibur_C17607_542*	98–104.81	5A	*Wsnp_Ex_C18107_26909127 - Wsnp_Ex_C1481_2831499*	198.63–205.24	0.09	< 0.001
	5A	*Wsnp_Ex_C18107_26909127 - Wsnp_Ex_C1481_2831499*	198.63–205.24	7A.2	*Kukri_C33036_348 - Rac875_C17000_731*	139.97–142.61	0.08	< 0.001
Location-year mean	4B	*Excalibur_C29141_864 - Excalibur_C17607_542*	98–104.81	5A	*Wsnp_Ex_C18107_26909127 - Wsnp_Ex_C1481_2831499*	198.63–205.24	0.11	< 0.001
	5A	*Wsnp_Ex_C3772_6866645 - Wsnp_Ex_C31672_40435001*	180.73–190.13	7D	*Ku_C17958_576 - Excalibur_C13094_523*	23.78–30.35	− 0.09	< 0.001
	7A.2	*Bs00063458_51 - Tplb0036a12_207*	156.82–157.34	7D	*Ku_C17958_576 - Excalibur_C13094_523*	23.78–30.35	− 0.06	0.015

**Fig. 3:**
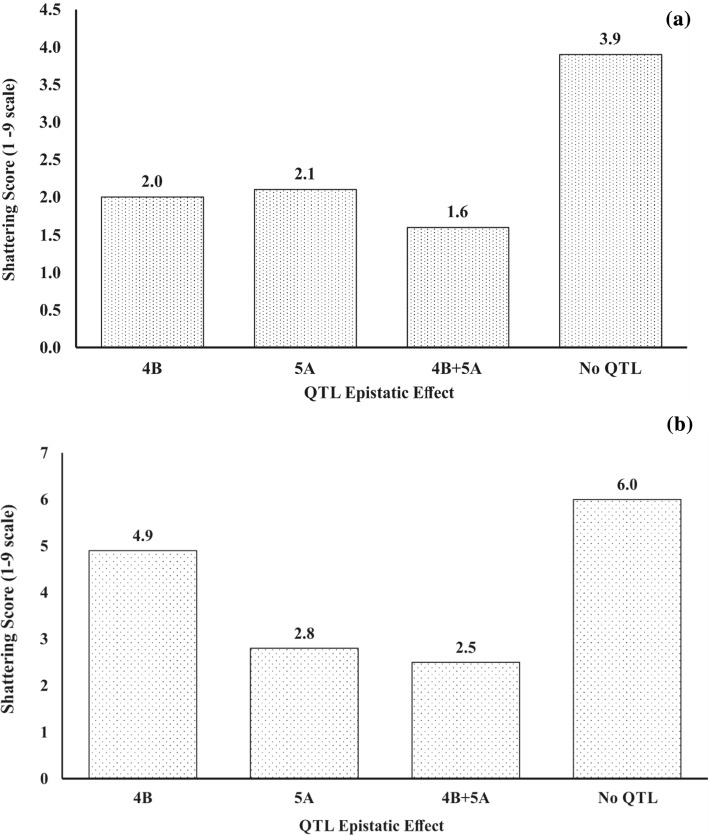
Examples of the additive x additive epistasis between *Sh.Sparc-4B* and *Sh.Sparc-5A* for shattering scores of the (**a**) Carberry/Thatcher population from Centre Farm in 2018; and (**b**) Carberry/AC Cadillac population from Centre Farm, near Swift Current in 2012. The epistatic interactions were determined using *Sh.Sparc-4B* QTL marker *IACX557* and *Sh.Sparc-5A* QTL marker *wsnp_Ex_c16715_25264080* in Carberry/Thatcher population, and between the *Sh.Sparc-4B* marker *wsnp_Ex_c21217_30347572* and *Sh.Sparc-5A* QTL marker *wsnp_Ex_c18107_26909127* in Carberry/AC Cadillac population

Epistatic interactions contributing to increased (A1 x A2 effect is negative) shattering in the Carberry/AC Cadillac population were: *Sh.Sparc-1A/Sh.Sparc-4B*, *Sh.Sparc-2D/Sh.Sparc-5A*, *Sh.Sparc-*3A*/Sh.Sparc-4B*, *Sh.Sparc-3A/Sh.Sparc-5A*, *Sh.Sparc-4B/Sh.Sparc-7D*, *Sh.Sparc-5A/Sh.Sparc-7D* and *Sh.Sparc-7A/Sh.Sparc-7D* (Table 4). Among these interactions, *Sh.Sparc-5A/Sh.Sparc-7D* was the most consistent appearing in four out of five environments. An example graphical depiction of individual effects in an epistatic interaction is presented in Fig. 3a and b between QTL *Sh.Sparc-4B* and *Sh.Sparc-5A* for the two populations and selected environments. For Carberry/Thatcher considering the Centre Farm 2018 trial, the shattering score was 3.9 when 4B and 5A resistance alleles were absent compared with 1.6 for the simultaneous presence of these QTL alleles (Fig. 3a). With the simultaneous occurrence of the 4B and 5A resistance alleles, the mean shattering score at the Centre Farm 2012 trial was 2.5 compared to 6.0 in the absence of both these alleles in the Carberry/AC Cadillac population (Fig. 3b). When the effects of individual QTL were compared, the score was 2.8 with the presence of 5A alone, and 4.9 with 4B alone. The presence of the 5A QTL alone resulted in a 2.1 shattering score and 2.0 for the 4B locus alone.

## Discussion

The continuous distribution of the response to spike shattering in the studied populations suggests polygenic inheritance of the shattering trait. The wide range of heritability values of the trait over environments and populations similarly suggests this trait is under complex genetic control with gene networks influenced by environment. Nevertheless, the broad sense heritability of the resistance to shattering observed in both populations indicated the opportunity to maintain a desirable expression of the trait through selection. The values of the environmental component as the converse to the broad sense heritable component similarly suggested the resistance to shattering is a complex trait. Previous studies indicate traits such as glume tenacity and kernel size coupled with environmental factors of wind and humidity can greatly influence the ability of a cultivar to hold its grain in a recoverable position for the period after maturity until harvest which can be several weeks (Clarke and DePauw 1983; Harrington and Waywell 1950). The moderate to moderately strong positive correlations (*P* < 0.0001) in the shattering scores observed among the environments is another indication of heritable genetic expression in the studied populations. Edaphic conditions have not been reported to influence seed shattering, although available soil moisture affects seed size and weight. Within a genotype, conditions that produce larger kernels place more stress on glumes (Chang 1943; Porter 1959). Damage by birds was not evident in any of the years. The range in maturity of the genotypes was about seven days. The variation in the correlations observed among shattering scores indicated the primary difference between environments, was likely wind speed after maturity. The average maximum daily wind speed for the period 1–10 September over the seven years varied from 24.2 to 32.4 km hr^−1^ (https://climate.weather.gc.ca/historical_data/search_historic_data_e.html).

The continuous nature of the phenotypic distributions is consistent with results of the QTL analysis which revealed multiple quantitative loci. This finding agrees with other studies that report multiple genes with quantitative control of spike shattering in wheat (Jantasuriyarat et al. 2004; Marza et al. 2006; Zhang and Mergoum 2007). With both parents of the Carberry/AC Cadillac population contributing positive and negative alleles for shattering, the apparent transgressive segregation of lines in two directions was expected. Transgressive segregation can occur because of the action of loci with complementary additive effects differentially present in parental lines combining in progeny (Rieseberg et al. 1999).

The observation that Thatcher was more resistant to shattering than Carberry, but that this resistance was not reflected in the results of the QTL mapping, with no resistance alleles attributed to Thatcher, is difficult to explain. Based on a shattering test conducted near Saskatoon, SK in 1948, Harrington and Waywell (1950) described Thatcher as a highly resistant wheat with a score of 1% compared with other cultivars such as Marquis at 2%, and Prelude at 23% shattering. Given no QTL for resistance was detected from Thatcher, it is possible several genes are present but lack sufficient expressivity to produce statistically significant effects on the phenotype. The other possibility could be sparse marker placement near genes making them undetected by QTL analysis or some combination of these two scenarios. That genetic differences occur between the two cultivars is supported by the occurrence of transgressive segregation in the progeny. There may also be loci not segregating between Thatcher and Carberry given the level of transgressive segregation appeared to be lower in the Carberry/Thatcher population than the Carberry/AC Cadillac population.

The shattering response of Carberry was significantly higher than that of Thatcher. However, the shattering resistance of Carberry is in large part due to the consistently and strongly expressed resistance alleles of the *Sh.Sparc-4B* and *Sh.Sparc-5A* QTL and minor alleles at *Sh.Sparc-7A.1* and *Sh.Sparc-7A.2*. Apart from the main effects, the desirable epistatic interactions detected between *Sh.Sparc-4B* and *Sh.Sparc-5A* across two populations and multiple environments contributed to Carberry’s shattering resistance. The *Sh.Sparc-5A* interaction with *Sh.Sparc-7A.2* in a single environment would have also contributed sporadically to Carberry’s shattering resistance. The year the interaction was discovered was a year Carberry expressed it’s highest level of resistance among the Carberry/AC Cadillac experiments.

The effect of the absence of Carberry resistance alleles is shown in its progeny by the most shattering susceptible lines of the Carberry/Thatcher population rated a relatively high seven out of nine. The QTL identified in the Carberry/Thatcher population were confirmed in the Carberry/AC Cadillac population, with additional QTL contributed by AC Cadillac. The wider distribution of Carberry/AC Cadillac compared to Carberry/Thatcher, with lines scoring as high as eight out of nine is consistent with the greater segregation of QTL in Carberry/AC Cadillac population.

The *Sh.Sparc-4B* chromosomal region not only is important in controlling shattering, but is an important genomic region for other agronomic traits such as yield, plant height, and disease resistance (Dhariwal et al. 2020; Duan et al. 2020; Garcia et al. 2019; Pandey et al. 2015; Toth et al. 2018)*.* In both populations, *Sh.Sparc-4B* was consistently associated with shattering and plant height in coupling phase. Using Ning7840/Clark wheat population, Marza et al (2006) reported two SSR markers, *Barc163* and *Barc20,* which mapped close to *Sh.Sparc-4B* markers based on the wheat consensus map of Bokore et al (2020). The two markers were not only associated with shattering resistance but increased grain yield and reduced plant height in Clark wheat. Peak QTL markers for *Sh.Sparc-4B, wsnp_BF482960B_Ta_1_4* and *Ex_c101685_705,* in the Carberry/AC Cadillac genetic map were, respectively, 2.27 cM and 2.07 cM from *Xbarc20*, and *Tdurum_contig42229_113* and *IAAV971* in the Carberry/Thatcher genetic map were each 0.12 cM from *Xbarc20* on the consensus map of Bokore et al (2020). Dhariwal et al (2020) reported that the same markers tagging the *Sh.Sparc-4B* Carberry QTL*, Ex_c101685_705* and *Tdurum_contig42229_113* are associated with plant height and Fusarium head blight deoxynivalenol (DON) response in the Canadian red spring wheat cultivar AAC Tenacious (Brown et al. 2015). The source of the Carberry QTL for height on chromosome 4BS is not entirely clear, but Carberry has been reported to have *Rht-B1b* (Pandey et al. 2015; Toth et al. 2018). Taller plants travel through a larger arc than shorter plants. The association of shattering and plant height might be a function of physical dynamics and not an association with properties of attachment of kernels and chaff parts in the spike per se.

Another *Sh.Sparc-4B* marker, *EX_C101685_705* was associated with grain weight, kernel length, kernel width, and kernel thickness in the Chinese wheat population Shannong 01–35/Gaocheng 9411 (Duan et al. 2020). Furthermore, the *Sh.Sparc-4B* marker *IAAV971* was associated with yield QTL *QYld.aww-4B* and *Rht-B1* in Australian wheat (Garcia et al. 2019). Likewise, *wsnp_BF482960B_Ta_1_4* was associated with a Septoria tritici blotch resistance QTL, *QStb.teagasc-4B.1,* that segregated in a winter wheat population (Riaz et al. 2020). Breeding and selection to bring the desirable alleles in this region into coupling would simplify multiple trait improvement through marker-assisted breeding in the future, as is the current situation of *Sh.Sparc-4B* controlling reduced shattering being in coupling with reduced plant height. A BLAST search of annotated genes in the region of *Sh.Sparc-4B* based on RefSeq v1.0 (IWGSC 2018) identified *TraesCS4B01G042300, TraesCS4B01G049800* and *TraesCS4B01G051900.* Xu et al. (2019) indicated that in the absence of *Rht-B1* due to a deletion, an interval of six genes including *TraesCS4B01G042300* may reduce plant height in wheat line Doumai. *TraesCS4B01G049800* was reported as a putative receptor protein kinase and a putative gene for grain yield within *QYLD.aww-4B* (Garcia et al. 2019). The effect of these genes on shattering is yet to be defined.

The markers associated with the second consistently expressed Carberry locus, *Sh.Sparc-5A,* reside in a similar region as a QTL on chromosome arm 5AL that consistently affected threshability traits in the W-7984/Opata 85 wheat population (Jantasuriyarat et al. 2004). The W-7984/Opata 85 threshability locus is believed to represent the free-threshing wheat gene *Q*. The marker *Xgwm126* for the 5AL QTL reported by Jantasuriyarat et al (2004) was located 10 cM from *Xwmc110* on the high-density SSR consensus map of Somers et al (2004). The marker *Xwmc110* was located only 0.6 cM from the *Sh.Sparc-5A* markers, *Kukri_rep_c102608_599* and *wsnp_Ex_c18107_26909127* in an SSR and SNP integrated map by Wen et al (2017)*.* The physical distance on the bread wheat reference genome sequence (IWGSC RefSeq v2.0) assembly of *Xwmc110* to *Kukri_rep_c102608_599* was 1.2 Mb, and it was 0.73 Mb from *Xwmc110* to *wsnp_Ex_c18107_26909127* (https://urgi.versailles.inrae.fr/blast_iwgsc/blast.php). The close proximity of markers between studies suggest the *Q* gene could be responsible for the *Sh.Sparc-5A* QTL. As all three cultivars are expected to have the *Q* gene, *Sh.Sparc-5A* might represent some subtle base pair difference. The broader interval observed in the *Sh.Sparc-5A* QTL region compared with that of *Sh.Sparc-4B,* and the association of the *Sh.Sparc-5A* with several markers is helpful in the development of diagnostic markers for marker-assisted breeding. Two high confidence genes *TraesCS5A01G463600* and *TraesCS5A01G465200* were located in the *Sh.Sparc-5A* shattering QTL region (IWGSC 2018)*.*

Another study (Marza et al. 2006) reported a shattering resistance QTL on chromosome 5A in the United States soft red winter wheat Clark, but it is different from the Carberry *Sh.Sparc-5A* QTL because the location of markers associated with the two QTL are too far apart. In the consensus map that integrates SNP and SSR markers (Bokore et al. 2020), QTL associated markers *Kukri_rep_c102608_599* for *Sh.Sparc-5A* and *Xbarc180* for the 5A threshability QTL of Clark were 118 cM from each other. The expression of the two QTL also suggests they are different with the 5A QTL in Clark (Marza et al. 2006) being highly inconsistent over environments compared to the consistent expression of *Sh.Sparc-5A*.

The *Sh.Sparc-5A* locus appears to hold a complex of genes controlling multiple traits. For example, the *Sh.Sparc-5A* marker *Kukri_rep_c102608_599* is in the interval of the QTL that increases seed weight and spike length in the Chinese wheat Zhou 8425B (Gao et al. 2015). An allele having a positive effect on the harvest index in another Chinese wheat also lies in this interval (Chen et al. 2019). Other studies in which the *Xwmc110* marker is involved include Fusarium head blight resistance in the Canadian durum wheat line DT696 (Singh et al. 2008), ear emergence in elite European winter wheat germplasm (Griffiths et al. 2009), and the pasta quality mixogram parameter time-to-peak (Zhang et al. 2008).

The two Carberry QTL, *Sh.Sparc-7A.1* and *Sh.Sparc-7A.2,* are different as markers associated with each QTL are located in different genomic regions. Furthermore, the QTL behaved differently. *Sh.Sparc-7A.1* associated markers are located on chromosome arm 7AS, whereas the *Sh.Sparc-7A.2* markers are on arm 7AL in the high-density SNP map by Wang et al (2014). Additionally, a physical distance of 448 Mb observed between markers of *Sh.Sparc-7A.1* and *Sh.Sparc-7A.2* in the bread wheat reference genome sequence (IWGSC RefSeq v2.0) suggests they are distinct loci (https://urgi.versailles.inrae.fr/blast_iwgsc/blast.php). The expression in only one out of four environments and marginally significant LOD score for *Sh.Sparc-7A.1* compared to the relatively stable QTL at *Sh.Sparc-7A.2* that expressed in three out of five environments supports the hypothesis that the two loci represent different genes. In addition to its consistency over environments, the epistasis of *Sh.Sparc-7A.2* with *Sh.Sparc-5A* resulting in reduced shattering compared with either locus alone makes *Sh.Sparc-7A.2* more appealing in breeding than *Sh.Sparc-7A.1*.

Based on the hexaploid wheat consensus map of Bokore et al (2020) that integrates SSR and SNP markers, markers for *Sh.Sparc-7A.2, Kukri_rep_c105157_485* and *wsnp_Ex_c19005_27918129*, were within 0.04–0.29 cM of *Xbarc108* that tagged a shattering resistance QTL in the Clark wheat cultivar (Marza et al. 2006). Additionally, *Xbarc108* is associated with grain protein (*QGpc.usw-A3*) with little effect on grain yield in Strongfield durum wheat (Suprayogi et al. 2009). The shattering resistance region *Sh.Sparc-7A.2* could be combined with grain protein (*QGpc.usw-A3*) using marker assisted selection. Zhang and Mergoum (2007) reported a major kernel shattering resistance QTL near the centromere of chromosome 7AL and a minor locus on the distal end of 7AL both of which were contributed by a hard red spring wheat cultivar Stoa. The map distance from *Sh.Sparc-7A.2* associated marker *wsnp_Ex_c19005_27918129* to *Xwmc633,* a marker associated with the minor 7AL QTL in Stoa was 108.5 cM (Wen et al. 2017), suggesting the region is different from *Sh.Sparc-7A.2*. Overlapping markers were not found to compare if the second Stoa 7A QTL was located in a similar region as either of the Carberry loci.

The low shattering QTL identified from AC Cadillac, *Sh.Sparc-7D* is located on chromosome arm 7DS. No QTL has been previously reported on 7DS, but a 7DL linkage group carries a shattering resistance factor that segregated in the Ning7840/Clark wheat population (Marza et al. 2006). The *Sh.Sparc-2D* QTL from AC Cadillac*,* located on chromosome arm 2DL, appears to be novel, although QTL for shattering resistance were reported on 2DS in the two different wheat populations W-7984/Opata 85 (Jantasuriyarat et al. 2004) and Ning7840/Clark (Li et al. 2016). Our report of the remaining AC Cadillac shattering resistance QTL located on 1AL, 3AL and 3DL appears to be a first. Markers associated with the 1AL and 3AL shattering resistance have been associated with other agronomic traits. For example, the 1AL QTL marker *Kukri_c58155_786* was associated with wheat proteins (Taranto et al. 2020). One of the markers which tagged the 3AL shattering resistance allele, *Wsnp_Ku_C44716_51926415,* is associated with flag leaf traits such as length, width, angle, and area (Wu et al. 2016), highlighting the importance of this region in trait improvement.

Results of the present study indicated that the additive genetic effect is a major component of heritability, although epistatic interactions contributed to a significant portion of the heritable variation which is consistent with other research findings (Ma et al. 2006; Zhou et al. 2017). The consistent detection of epistasis between the two major QTL *Sh.spa-4B* and *Sh.spa-5A* in the present study contrasts with the sporadic occurrences of interactions between major and minor effect QTL. According to Zhou et al (2017), significant epistasis is possible between QTL that individually have low phenotypic effects, but no epistasis was detected between minor QTL in our study. The epistatic interactions between pairs of Carberry alleles *Sh.spa-4B / Sh.spa-5A* and *Sh.spa-5A / Sh.spa-7A.2* are desired for improving shattering resistance. This reduction in shattering can be illustrated by results of the Centre Farm 2012 trial that involved the Carberry/AC Cadillac population, among other examples. Similar favorable epistatic combinations are likely to be common because breeders select lines with reduced-shattering while retaining threshability. Conversely, the increased level of shattering observed with the remaining digenic interactions that involved the 4B or 5A with the QTL from AC Cadillac suggest caution may be needed when planning crosses to take into account unfavorable combinations of loci.

In summary, the shattering trait showed intermediate heritability with medium to high correlations observed between the scores in different environments. Nine main effect QTL were identified from Carberry and AC Cadillac using MapQTL that demonstrated the complex inheritance of the shattering trait. Despite having low shattering scores compared to Carberry, no QTL were detected from the heritage cultivar Thatcher, likely due to the lack of sufficient expressivity of QTL or sparse marker placement near shattering genes or a combination of these two scenarios. Of the nine QTL we identified, four desirable Carberry alleles were located on chromosome arms 4BS, 5AL, 7AS and 7AL, and five QTL desirable AC Cadillac alleles were located on 1AL, 2DL, 3AL, 3DL and 7DS. The QTL on 4BS and 5AL with consistent expression across populations and environments are major QTL responsible for the control of seed shattering. The 4B QTL was consistently associated with reduced shattering and reduced plant height in coupling phase. Based on proximity, there might be some modification to the *Q* gene that may be responsible for the 5AL QTL, as the three cultivars would have the *Q*-gene. The two remaining Carberry QTL and the other five AC Cadillac loci represent minor QTL having weak and variable expressions across environments. Analysis by QTLNetwork demonstrated the importance of epistasis with nine significant additive x additive epistatic interactions between main effect loci. The interactions between main effect QTL *Sh.Sparc-4B* and *Sh.Sparc-5A,* and between *Sh.Sparc-5A* and *Sh.Sparc-7A.2* are synergistic and thus beneficial in breeding for improved shattering resistance. In contrast, the other seven pairs of interacting QTL *Sh.Sparc-1A/4B*, *Sh.Sparc-2D/5A*, *Sh.Sparc-*3A*/4B*, *Sh.Sparc-3A/5A*, *Sh.Sparc-4B/7D*, *Sh.Sparc-5A/7D* and *Sh.Sparc-7A/7D* were detrimental by increasing the expression of shattering. SNP markers closely associated with the QTL will be helpful in characterizing parents and for the identification of detrimental alleles and combinations of alleles across loci for culling early generation breeding lines.

## Supplementary Information

Below is the link to the electronic supplementary material.

Supplementary file1 (DOCX 15 kb)

## Data Availability

All phenotypic and sequence data generated and used in this study have been deposited in The Crop Information Engine and Research Assistant (CIERA), Agriculture and Agri-Food Canada.
