# Supplementary material for: Main effect and epistatic QTL affecting spike shattering and association with plant height revealed in two spring wheat (Triticum aestivum L.) populations
Source: Theor Appl Genet. 2022 Mar 20;135(4):1143–62. doi: 10.1007/s00122-021-03980-2 (PMC9033718; doi:10.1007/s00122-021-03980-2)
Supplement: Supplementary file 1 — Supplementary file1 (DOCX 15 kb) [file 122_2021_3980_MOESM1_ESM.docx]

**Supplemental Table 1** Statistics for the high density genetic maps of Carberry/Thatcher and Carberry/AC Cadillac populations.

| **Linkage group** | **Number of markers** | **Length, cM** | **Map density (cM/marker)** | **Biggest interval, cM** |
| --- | --- | --- | --- | --- |
| **A) Carberry/Thatcher population** |  |  |  |  |
| 1A | 572 | 238 | 0.4 | 10.8 |
| 1B part 1 | 390 | 142.9 | 0.4 | 22.9 |
| 1B part 2 | 92 | 16.1 | 0.2 | 2.1 |
| 1D | 76 | 74.5 | 1 | 20.3 |
| 2A | 376 | 190.4 | 0.5 | 22.6 |
| 2B | 1300 | 357.1 | 0.3 | 27.7 |
| 2D | 38 | 93.6 | 2.5 | 23.1 |
| 3A | 474 | 243.4 | 0.5 | 23.9 |
| 3B part 1 | 107 | 110.4 | 1 | 28 |
| 3B part 2 | 58 | 24 | 0.4 | 2.8 |
| 3D part 1 | 136 | 135.5 | 1 | 21.3 |
| 3D part 2 | 109 | 5.5 | 0.1 | 1.7 |
| 4A part 1 | 194 | 79.6 | 0.4 | 8 |
| 4A part 2 | 228 | 25.1 | 0.1 | 10.3 |
| 4B | 403 | 172.4 | 0.4 | 14 |
| 5A | 485 | 253.8 | 0.5 | 20.7 |
| 5B | 831 | 323.6 | 0.4 | 21.4 |
| 5D part 1 | 129 | 44.8 | 0.4 | 13.2 |
| 5D part 2 | 31 | 15 | 0.5 | 4.8 |
| 5D part 3 | 5 | 6.4 | 1.7 | 5.2 |
| 6A | 301 | 180.1 | 0.6 | 25.7 |
| 6B | 689 | 272.6 | 0.4 | 37.1 |
| 6D part 1 | 84 | 32.4 | 0.4 | 11.5 |
| 6D part 2 | 43 | 8.3 | 0.2 | 2.7 |
| 7A | 678 | 279 | 0.4 | 23.5 |
| 7B | 355 | 128.2 | 0.4 | 11.2 |
| 7B part 2 | 106 | 70.6 | 0.7 | 15.9 |
| 7D | 70 | 122.9 | 1.8 | 25.3 |
| **Total** | **8360** | **3645.8** | **0.6** |  |
| **B) Carberry/AC Cadillac population** |  |  |  |  |
| 1A | 537 | 213.9 | 0.4 | 12.6 |
| 1B | 635 | 187.3 | 0.29 | 32.1 |
| 1D | 96 | 110.3 | 1.15 | 26.6 |
| 2A | 214 | 201.1 | 0.94 | 24.3 |
| 2B | 794 | 191.7 | 0.24 | 16.5 |
| 2D part 1 | 48 | 100.8 | 2.1 | 27.3 |
| 2D part 2 | 148 | 23.1 | 0.16 | 15.1 |
| 3A | 351 | 218.1 | 0.62 | 19 |
| 3B part 1 | 45 | 48.7 | 1.08 | 19 |
| 3B part2 | 122 | 41.3 | 0.34 | 10.7 |
| 3B part3 | 59 | 12.6 | 0.21 | 6.8 |
| 3D part 1 | 36 | 17.8 | 0.49 | 7.4 |
| 3D part2 | 11 | 28.1 | 2.56 | 7.7 |
| 3D part3 | 65 | 3.7 | 0.06 | 1.5 |
| 4A part 1 | 175 | 114.3 | 0.65 | 21.5 |
| 4A part2 | 78 | 15.6 | 0.2 | 4 |
| 4B | 306 | 160.5 | 0.52 | 12.2 |
| 4D | 17 | 10 | 0.59 | 4.3 |
| 5A | 434 | 250.7 | 0.58 | 23.8 |
| 5B | 564 | 252.3 | 0.45 | 21.4 |
| 5D part 1 | 124 | 85 | 0.69 | 19.1 |
| 5D part2 | 21 | 10 | 0.47 | 4.2 |
| 6A | 324 | 150.6 | 0.46 | 21.6 |
| 6B | 566 | 237.9 | 0.42 | 17.6 |
| 6D | 92 | 82.4 | 0.9 | 33.3 |
| 7A | 685 | 241.2 | 0.35 | 17.6 |
| 7B part 1 | 178 | 97.5 | 0.55 | 12.4 |
| 7B part2 | 43 | 60.5 | 1.41 | 26.2 |
| 7D | 38 | 71 | 1.87 | 21.6 |
| Total | 6806 | 3237.9 | 0.72 | - |
